# *N*-Substituted 5–Chloro-6-phenylpyridazin-3(2*H*)-ones: Synthesis, Insecticidal Activity Against *Plutella xylostella* (L.) and SAR Study

**DOI:** 10.3390/molecules17089413

**Published:** 2012-08-06

**Authors:** Jian Wu, Shenghong Kang, Qinkun Yuan, Lijun Luo, Juan Ma, Qingcai Shi, Song Yang

**Affiliations:** State Key Laboratory Breeding Base of Green Pesticides and Agricultural Bioengineering, Key Laboratory of Green Pesticides and Agricultural Bioengineering, Ministry of Education, Guizhou University, Guiyang 550025, China; Email: concenhom0627@126.com (S.K.); momo1989.ok@163.com (Q.Y.); luolijun01013610@163.com (L.L.); majuanmalong@163.com (J.M.); shiqingcai1988@yahoo.com.cn (Q.S.); yangsdqj@126.com (S.Y.)

**Keywords:** pridazin-3(2*H*)-one, synthesis, insecticidal activity, SAR study, *Plutella xylostella*

## Abstract

A series of *N*-substituted 5–chloro-6-phenylpyridazin-3(2*H*)-one derivatives were synthesized based on our previous work; all compounds were characterized by spectral data and tested for *in vitro* insecticidal activity against *Plutella xylostella*. The results showed that the synthesized pyridazin-3(2*H*)-one compounds possessed good insecticidal activities, especially the compounds **4b**, **4d**, and **4h **which showed >90% activity at 100 mg/L. The structure-activity relationships (SAR) for these compounds were also discussed.

## 1. Introduction

The diamondback moth (*Plutella xylostella* L.) is a serious pest insect in many parts of the World [1,2]. Serious yield losses to crucifers (such as cabbage, cauliflower, broccoli, brussels sprouts and turnip) from the diamondback moth have become more common in recent years. Currently, insecticide application is the primary method for controlling this pest [2,3], but particularly severe diamondback moth resistance to insecticides has been resulting from the indiscriminate use of pesticides in many tropical and subtropical countries [4,5,6] so controlling the diamondback moth has become more and more difficult, and the development of novel insecticides for this insect has attracted more and more attention.

**Figure 1:**
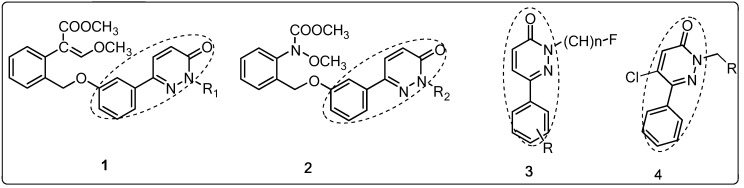
The common cores of biologically active compounds **1** to **4**.

**Scheme 1:**
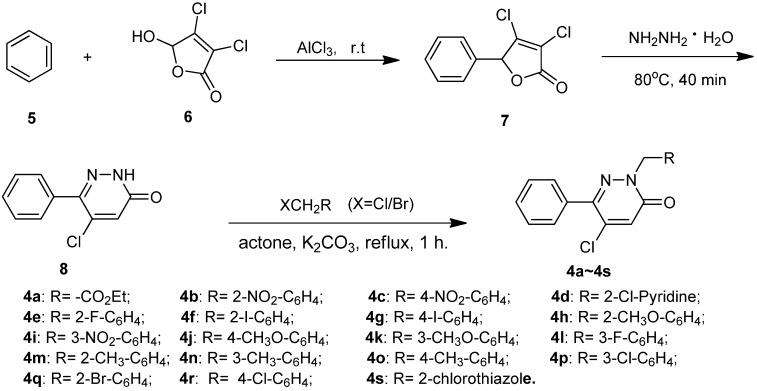
Synthetic route to *N*-substituted 5–chloro-6-phenylpyridazin-3(2*H*)-ones **4a**–**s**.

Pyridazinones, an important class of heterocyclic ring, have attracted more and more attention due to their broad-spectrum biological activity as plant virucides [7,8], antitumor agents [9], fungicides [10,11,12], insecticides [13,14], and herbicides [15,16,17]. In the insecticidal area, many pyridazin-3(2*H*)-one derivatives with good insecticidal activity have been discovered and commercialized, such as pyridaphenthion, pyidaben, NC-184, and NC-170 [18]. Moreover, some compounds containing *N*-substituted 6-phenylpyridazin-3(2*H*)-one moieties (Figure 1, **1**–**3**) were reported, all of which showed excellent insecticidal activity against *Spodoptera exigua* (H.), *Heliothis* *virescens*, *Tetranychus urticae*, *et al.* [19,20,21]. In our previous study, several *N*-substituted 6-phenylpyridazin-3(2*H*)-one derivatives **4** were reported, which showed fungicidal activity against *G. zeae*, *F. oxysporum* and *C. mandshurica* to a certain extent [22]. However, in the process of developing novel insecticidal molecules, we noted that these compounds **1**–**4** have as a common structure (*N*-substituted 5–chloro-6-phenylpyridazin-3(2*H*)-one), see Figure 1). With this in mind, in an effort to discover new scope of application for compounds **4**, we sought to test their insecticidal activity against *Plutella xylostella* (L.), and occasionally found that some of the pyridazin-3(2*H*)-one derivatives [22] showed 100% insecticidal activity against *Plutella xylostella* at 100 mg/L. In the current work, several new *N*-substituted 5–chloro-6-phenylpyridazin-3(2*H*)-one derivatives **4i**–**s** were synthesized based on our previous synthetic route (Scheme 1) [22], the insecticidal activity and structure-activity relationship (SAR) for these compounds [including the compounds (**4a**–**h**) in reference [22] against *Plutella xylostella* were evaluated and discussed, respectively.

## 2. Results and Discussion

### 2.1. Chemistry

The synthetic protocols of the title compounds was depicted in Scheme 1. Friedel-Crafts alkylation of benzene with mucochloric acid leads to *γ*-phenyldichlorocrotonolactone (**7**), then **7** reacts in a complex manner with hydrazine hydrate, with elimination of one atom of chlorine [23], to afford a high yields of 5–chloro-6-phenylpyridazin-3(2*H*)-one (**8**). Compounds **4a**–**s** were then obtained in excellent yields by reaction of **8** with different halides based on our previous work [22]. The structures of the synthesized compounds were confirmed by ^1^H-NMR, ^13^C-NMR, IR and elemental analysis. All spectral and analytical data were consistent with the assigned structures.

### 2.2. Insecticidal Activity

As indicated in Table 1, many of the synthesized compounds exhibited weak to excellent insecticidal activities against *P. xylostella* at 100 mg/L. Compounds **4b**, **4d**, **4h** and **4t** showed 100%, 93%, 97% and 84% activity at 100 mg/L, respectively. Compounds **4f**, **4g** and **4l** showed moderate activities against *P. xylostella* at 100 mg/L (50%, 60% and 62%, respectively). When the concentration was 50 mg/L, compound **4b** still showed 97% activity against *P. xylostella*, which was similar as that of chlorpyrifos (97%), and compound **4s** possessed 84% activity on *P. xylosella*. In addition, compounds **4d** and **4h **also displayed >70% activity on *P. xylostella* at 50 mg/L. Moreover, when the concentration was 25 mg/L, the insecticidal activities were decreased, although we noted that compounds**4b**, **4d**, **4h** and **4s** still possessed insecticidal activity to a certain extent (21%, 20%, 20% and 15%, respectively).

### 2.3. Structure-Activity Relationship (SAR) Study

The preliminary SAR analysis indicated that a big (in bulk) group with strong electronegativity at the 2-postion on the benzene (in a –CH_2_R group) has a positive influence enhancing the insecticidal activity of the synthesized compounds, that’s why the compounds **4b** (–CH_2_R = 2-NO_2_-C_6_H_4_CH_2_-) and **4h** (–CH_2_R = 2–CH_3_O-C_6_H_4_CH_2_-) showed higher activity than **4e** (–CH_2_R = 2-F-C_6_H_4_CH_2_-) and **4m** (–CH_2_R = 2–CH_3_-C_6_H_4_CH_2_-), therefore, we can speculate that both the bulk and electronegativity of the substituent group at the 2-position on benzene play important roles in the insecticidal activity against *P. xylostella*. In addition, the position of the group on benzene also a key factor for the activity, as we can see that compound **4b** showed excellent activity, while compound **4c** (–CH_2_R= 3-NO_2_-C_6_H_4_CH_2_-) and **4i** (–CH_2_R=4-NO_2_-C_6_H_4_CH_2_-) showed little (or no) activity (**4b** >**4c** > **4i**); a similar case can be found when the group was CH_3_O- (**4h** > **4j** > **4k**). Moreover, the introduction of 2-Cl-substituted pyridine and thiazole in group of –CH_2_R can also enhance insecticidal activity, e.g., compound **4b** (–CH_2_R = 2-Cl-pyridine–CH_2_-) and **4t** (–CH_2_R = 2-Cl-thiazole–CH_2_-) also displayed good insecticidal activity against *P. xylostella*.

**Table 1:** Insecticidal activity of the synthesized compounds against *P. xylostella*.

Comp.	Insecticidal activity (%) at a concentration of (mg/L)		
	100	50	25
**4a**	20	/	/
**4b**	**100**	**97**	21
**4c**	25	0	/
**4d**	**93**	**76**	20
**4e**	13	/	/
**4f**	50	13	0
**4g**	45	0	/
**4h**	**97**	**70**	20
**4i**	10	0	/
**4j**	45	13	0
**4k**	60	30	13
**4l**	62	33	0
**4m**	20	0	/
**4n**	15	/	/
**4o**	14	/	/
**4p**	21	0	/
**4q**	43	10	/
**4r**	31	0	/
**4s**	**84**	**60**	15
Blank control	0	0	0
Chlorpyrifos	100	97	67
Avermectin	100	100	100

## 3. Experimental

### 3.1. Chemistry

Melting points were determined by using a XT-4 binocular microscope (Beijing Tech Instrument Co., Beijing, China) and are uncorrected. ^1^H and ^13^C-NMR spectra were recorded on a JEOL ECX 500 NMR spectrometer operating at room temperature and 500 MHz using acetone-*d*_6_ or CDCl_3_ as solvent and TMS as an internal standard. Infrared spectra were recorded by KBr using a Bruker VECTOR 22 spectrometer. Elemental analysis was performed using an Elemental Vario-III CHN analyzer. The course of the reactions was monitored by TLC; analytical TLC was performed on silica gel GF254. All reagents were of analytical grade or chemically pure. All anhydrous solvents were dried and purified according to standard techniques just before use. All the intermediates and title compounds were prepared according to the literature [22]. The properties for compounds **4a**–**h** were reported in our previous work [22]. The properties for **4i**–**s** are listed as follows. 

*5–Chloro-2-(3-nitrobenzyl)-6-phenylpyridazin-3(2H)-one* (**4i**): White solid; yield: 76%; m.p.: 79–80 °C; ^1^H-NMR (CDCl_3_) *δ*: 8.32 (s, 1H, Ph-H), 8.18, (*J* = 8.0 Hz, 1H, Ph-H), 7.81 (1H, *J* = 7.45 Hz, Ph-H), 7.47–7.56 (m, 6H, 6Ph-H), 7.15 (s, 1H, pyridazine-H), 5.43 (s, 2H, CH_2_); ^13^C-NMR (CDCl_3_) *δ*: 158.71, 148.49, 145.90, 140.21, 137.51, 135.12, 133.31, 129.82, 129.21, 129.02, 128.42, 123.83, 123.33, 54.61, 49.73; IR (KBr): *ν* 3058.0, 2951.0, 2834.1, 1673.6 cm^−1^; Anal. Calc. for C_17_H_12_ClN_3_O_3_: C 59.75, H 3.54, N 12.30. Found: C 59.69, H 3.60, N 12.33.

*5-Chloro-2-(4-methoxybenzyl)-6-phenylpyridazin-3(2H)-one* (**4j**): White solid; yield: 78%; m.p.: 138–140 °C; ^1^H-NMR (CDCl_3_) *δ*: 8.22 (d, ^3^*J* = 8.6 Hz, 2H, 2Ph-H), 7.63 (d, ^3^*J* = 8.6 Hz, 2H, 2Ph-H) 7.43–7.52 (m, 5H, 5Ph-H), 7.12 (s, 1H, pyridazine-H), 5.43 (s, 2H, CH_2_), 3.85 (s, 3H, OCH_3_); ^13^C-NMR (CDCl_3_) *δ*: 158.72,148.44, 145.91, 141.22 133.53, 132.16, 133.33, 128.82, 127.28, 127.08, 126.43, 122.88, 122.33, 53.89, 49.74; IR (KBr): *ν* 3016.2, 2961.3, 1672.6 cm^−^^1^ Anal. Calc. for C_18_H_15_ClN_2_O_2_: C 66.16, H 4.63, N 8.57. Found: C 66.19, H 4.60, N 8.61.

*5-Chloro-2-(3-methoxybenzyl)-6-phenylpyridazin-3(2H)-one* (**4k**): White solid; yield: 76%; m.p.: 83–85 °C; ^1^H-NMR (CDCl_3_) *δ*: 7.82 (s, 1H, Ph-H), 7.64, (d, *J *= 7.45 Hz, 1H, Ph-H), 7.53–7.55 (m, 2H, Ph-H), 7.45–7.47 (m, 2H, Ph-H), 7.41 (d, *J *= 7.45 Hz, 1H, Ph-H), 7.12 (d, 1H, pyridazine-H), 7.06 (t, *J *= 7.4 Hz, 1H, Ph-H), 5.27 (s, 2H, CH_2_), 3.82 (s, 3H, OCH_3_); ^13^C-NMR (acetone-*d*_6_) *δ*:158.38, 157.32, 144.47, 139.10, 134.23, 129.32, 128.80, 128.46, 128.14, 124.60, 110.50, 55.12, 49.81; IR (KBr): *ν* 3058.0, 2951.0, 2834.1, 1673.6 cm^−1^; Anal. Calc. for C_18_H_15_ClN_2_O_2_: C 66.16, H 4.63, N 8.57. Found: C 66.21, H 4.58, N 8.58.

*5-Chloro-2-(3-fluorobenzyl)-6-phenylpyridazin-3(2H)-one* (**4l**): White solid; yield: 73%; m.p.: 82–84 °C; ^1^H-NMR (CDCl_3_) *δ*:8.32 (t, *J* = 1.7 Hz, 1H, Ph-H), 8.18 (d, *J* = 8.6 Hz, 1H, Ph-H), 7.80 (d, *J* = 7.45 Hz, 1H, Ph-H), 7.46–7.56 (m, 6H, 6Ph-H), 7.15 (s, 1H, pyridazine-H), 5.44 (s, 2H, CH_2_); ^13^C-NMR (CDCl_3_) *δ*:157.75, 147.46, 144.95, 140.23, 136.53, 135.14, 133.23, 129.85, 129.72, 129.07, 128.45, 123.94, 123.33, 56.63; IR (KBr): *ν* 3058.5, 2955.3, 2835.5, 1675.6 cm^−1^; Anal. Calc. for C_17_H_12_ClFN_2_O: C 64.87, H 3.84, N 8.90. Found: C 64.89, H 3.86, N 8.88.

*5-Chloro-2-(2-methylbenzyl)-6-phenylpyridazin-3(2H)-one* (**4m**): White solid; yield: 78%; m.p.: 78.6–79.8 °C; ^1^H-NMR (acetone-*d*_6_) *δ*: 7. 7.37–7.87 (m, 9H, Ph-H), 7.15 (s, 1H, pyridazine-H), 5.45 (s, 2H, CH_2_), 2.35 (s, 3H, CH_3_); ^13^C-NMR (acetone-*d*_6_)*δ*: 156.35, 154.41, 146.47, 137.01, 133.29, 128.28, 128.36, 127.85, 127.45, 127.16, 122.30, 120.31, 108.67, 52.03, 20.12; IR (KBr): *ν* 3041.0, 2956.0, 2834.4, 1674.6 cm^−1^; Anal. Calc. for C_18_H_15_ClN_2_O: C 69.57, H 4.86, N 9.01. Found: C 69.62, H 4.88, N 8.98.

*5-Chloro-2-(3-methylbenzyl)-6-phenylpyridazin-3(2H)-one* (**4n**): White solid; yield: 78%; m.p.: 95.4–96.8 °C; ^1^H-NMR (acetone-*d*_6_)*δ*: 7.40–8.07 (m, 9H, Ph-H), 7.19 (s, 1H, pyridazine-H), 5.38 (s, 2H, CH_2_), 2.29 (s, 3H, CH_3_); ^13^C-NMR (acetone-*d*_6_)*δ*: 157.35, 154.45, 145.15, 136.61, 134.24, 128.56, 128.16, 127.55, 127.35, 127.13, 121.33, 120.33, 108.47, 52.83, 20.72; IR (KBr): *ν* 3045.1, 2955.6, 2837.6, 1665.6 cm^−1^; Anal. Calc. for C_18_H_15_ClN_2_O: C 69.57, H 4.86, N 9.01. Found: C 69.49, H 4.81, N 9.03.

*5-Chloro-2-(4-methylbenzyl)-6-phenylpyridazin-3(2H)-one* (**4o**): White solid; yield: 83%; m.p.: 99–101 °C; ^1^H-NMR (acetone-*d*_6_) *δ*: 7.93(d, ^3^*J *= 8.6 Hz, 2H, 2Ph-H), 7.43 (d, ^3^*J* = 8.6 Hz, 2H, 2Ph-H) 7.23–7.52 (m, 5H, 5Ph-H), 7.13 (s, 1H, pyridazine-H), 5.54 (s, 2H, CH_2_), 2.17 (s, 3H, CH_3_); ^13^C-NMR (acetone-*d*_6_) *δ*: 158.36, 156.45, 144.16, 135.65, 134.27, 128.76, 128.56, 126.56, 126.35, 126.13, 120.33, 120.30, 108.77, 57.83, 19.02; IR (KBr): *ν* 3044.8, 2945.5, 2834.6, 1668.4 cm^−1^; Anal. Calc. for C_18_H_15_ClN_2_O: C 69.57, H 4.86, N 9.01. Found: C 69.56, H 4.79, N 9.02.

*5-Chloro-2-(3–chlorobenzyl)-6-phenylpyridazin-3(2H)-one* (**4p**): Light yellow solid; yield: 83%; m.p.: 98–99 °C; ^1^H-NMR (acetone-*d*_6_) *δ*: 7.83 (s, 1H, Ph-H), 7.65 (d, *J *= 8.6 Hz, 1H, Ph-H), 7.45–7.56 (m, 5H, 5Ph-H), 7.43 (d, *J *= 8.6 Hz, 1H, Ph-H), 7.12 (s, 1H, pyridazine-H), 5.31 (s, 2H, CH_2_); ^13^C-NMR (acetone-*d*_6_) *δ*: 158.53, 144.03, 137.84, 137.38, 133.54, 130.48, 129.73, 129.32, 129.05, 128.37, 128.31, 94.55, 54.63; IR (KBr): *ν* 3044.7, 3024.4, 1664.5 cm^−1^; Anal. Calc. for C_17_H_12_Cl_2_N_2_O: C 61.65, H 3.65, N 8.46. Found: C 61.59, H 3.68, N 8.50.

*2-(2-Bromobenzyl)-5–chloro-6-phenylpyridazin-3(2H)-one* (**4q**): Light yellow solid; yield: 74%; m.p.: 79.5–81.2 °C; ^1^H-NMR (acetone-*d*_6_) *δ*: 8.08 (dd, *J*_1_ = 1.15, *J_2_*= 8.55 Hz, 1H, Ph-H), 7.44–7.58 (m, 7H, Ph-H), 7.19 (t, *J*=7.45 Hz, 1H, Ph-H), 7.17 (s, 1H, pyridazine-H), 5.77 (s, 2H, CH_2_); ^13^C-NMR (acetone-*d*_6_) *δ*: 158.9, 148.70, 146.02, 140.31, 133.78, 133.23, 131.08, 129.81, 129.28, 129.24, 129.07, 128.91, 128.41, 125.41, 52.60; IR (KBr): *ν* 3054.7, 3024.6, 1674.5 cm^−1^; Anal. Calc. for C_17_H_12_BrClN_2_O: C 54.35, H 3.22, N 7.46. Found: C 54.40, H 3.25, N 7.50.

*5-Chloro-2-(4–chlorobenzyl)-6-phenylpyridazin-3(2H)-one* (**4r**): White solid; yield: 86%; m.p.: 124–126 °C; ^1^H-NMR (CDCl_3_) *δ*: 7.66 (d, ^3^*J *= 8.6 Hz, 2H, 2Ph-H), 7.45–7.55 (m, 5H, 5Ph-H), 7.21 (d, ^3^*J* = 8.6 Hz, 2H, 2Ph-H), 7.11 (s, 1H, pyridazine-H), 5.28 (s, 2H, CH_2_); ^13^C-NMR (CDCl_3_) *δ*: 158.67, 145.61, 139.83, 137.90, 135.29, 133.55, 131.05, 129.69, 129.31, 128.80, 128.40, 94.19, 54.80; IR (KBr): *ν* 3045.7, 3023.4, 1664.6 cm^−1^; Anal. Calc. for C_17_H_12_Cl_2_N_2_O: C 61.65, H 3.65, N 8.46. Found: C 61.63, H 3.65, N 8.41.

*5-Chloro-2-((2–chlorothiazol-5-yl)methyl)-6-phenylpyridazin-3(2H)-one* (**4s**): Light yellow solid; yield: 78%; m.p.: 76–78 °C; ^1^H-NMR (acetone-*d*_6_) *δ*: 7.53–7.97 (m, 4H, Ph-H), 7.22 (s, 1H, Pyridazine-H), 6.72 (s, 1H, Thiazole-H), 5.25 (s, 2H, CH_2_); ^13^C-NMR (acetone-*d*_6_) *δ*: 156.37, 145.76, 137.67, 136.49, 135.78, 135.46, 127.95, 127.65, 127.32, 126.96, 126.63, 126.33, 125.53, 93.64, 53.35; IR (KBr): *ν* 3035.3, 3022.6, 1673.5 cm^−1^; Anal. Calc. for C_14_H_9_Cl_2_N_3_OS: C 49.72, H 2.68, N 12.42. Found: C 49.75, H 2.64, N 12.38.

### 3.2. Insecticidal Bioassays

The insecticidal activities for the synthesized compounds against *P. xylostella* were evaluated using previously reported procedures [24,25,26]. Fresh cabbage discs (diameter 2 cm) were dipped into the prepared solutions containing compounds **4a** to **4s** for 10 s, dried in air and placed in a Petri dish (diameter 9 cm) lined with filter paper. Ten larvae of second-instar *P. xylostella* were carefully transferred to the Petri dish. Avermectin and chlorpyrifos were used as controls; three replicates were performed for each experiment. Mortalities were determined after 72 h. The results were summarized in Table 1.

## 4. Conclusions

In the present study, a series of *N*-substituted 5–chloro-6-phenylpyridazin-3(2*H*)-one derivatives were synthesized by employing mucochloric acid and benzene as the starting materials. The synthesized compounds were characterized by spectral data (^1^H-NMR, ^13^C-NMR, IR) and elemental analysis. The compounds were tested for insecticidal activity *in vitro* against *P. xylostella*. The results showed that the synthesized pyridazin-3(2*H*)-one compounds possessed weak to good insecticidal activities, especially the compounds **4b**, **4d**, and **4h** whichshowed >90% activities at 100 mg/L. The preliminary SAR analysis indicated that a big (in bulk) group with strong electronegativity at the 2-postion on the benzene ring (in a –CH_2_R group) had a positive influence enhancing the insecticidal activity of the synthesized compounds; moreover, the introduction of a 2-Cl-substituted pyridine and thiazole in the –CH_2_R group can also enhance the insecticidal activity. Further studies are currently underway to optimize the structure to obtain better insecticidal activity in these *N*-substituted pyridazin-3(2*H*)-one derivatives.
